# Biorefinery and sustainability for the production of biofuels and value-added products: A trends analysis based on network and patent analysis

**DOI:** 10.1371/journal.pone.0279659

**Published:** 2023-01-12

**Authors:** Alejandro Barragán-Ocaña, Humberto Merritt, Omar Eduardo Sánchez-Estrada, José Luis Méndez-Becerril, María del Pilar Longar-Blanco

**Affiliations:** 1 Instituto Politécnico Nacional (IPN), Centro de Investigaciones Económicas, Administrativas y Sociales, Mexico City, Mexico; 2 Universidad Autónoma del Estado de México (UAEM), Centro Universitario UAEM Valle de Chalco, Valle de Chalco, State of Mexico, Mexico; Karl-Franzens-Universitat Graz, AUSTRIA

## Abstract

Biorefineries are modern mechanisms used for producing value-added products and biofuels from different biomass sources. However, a crucial challenge is to achieve a sustainable model for their adequate implementation. Challenges related to technical efficiency and economic feasibility are two of the most relevant problems. Therefore, the present study sought to determine the current trends in basic research and technological development around biorefining and sustainability. We carried out a co-occurrence analysis and a patent analysis using data obtained from the Scopus and Lens databases to provide a general overview of the current state of this area of knowledge. The co-occurrence analysis intends to provide an overview of biorefining and sustainability based on terms associated with these two concepts as a starting point to determine the progress and existing challenges of the field. The results of the patent analysis consisted in identifying the main technological sectors, applicants, and territories where inventions associated with biorefining are registered. The analysis of the information showed that bioeconomy, techno-economic aspects, circular economy, technical issues associated with biomass production, and biofuels represent the focal point of basic research in a wide range of disciplines. Technology development is focused on fermentation, enzymes, and microorganisms, among other areas, which shows the validity of these traditional techniques in addressing the problems faced by the bioeconomy. This scenario shows that developed economies are the driving force behind this area of knowledge and that the PCT system is fundamental for the protection and commercialization of these inventions in places different from where they originated. Furthermore, the challenge lies in learning to work in alternative and complementary technological sectors, beyond microbiology and enzyme applications, in pursuit of the sector’s technical and economic feasibility.

## Introduction

The term bioeconomy is associated with certain aspects of industrial competition and sustainability that have favorable effects on the environment; the European Union (EU) is responsible for some of the most remarkable advances in this field [1]. In the context of EU, industrial biotechnology represents a strategic tool to develop competition and sustainability, create jobs, and generate income within the bioeconomy framework; it also contributes to creating value chains and is used to transform waste into valuable resources (circular economy) [2]. Likewise, there is worldwide interest in producing second-generation biofuels using lignocellulosic biomass, although these are not yet a competitive market alternative [3]. Biorefinery plays a fundamental and innovative role in the circular bioeconomy, which promises both environmental and economic benefits. However, to achieve a sustainable scheme and guarantee profitable operating mechanisms, it is essential to increase the efficiency with which inputs are processed [4, 5]. Therefore, research is needed to understand the trends in both basic research and technological applications to determine the challenges and advances in biorefining as a tool for promoting sustainable development.

Thus, in a circular economy, biologically-based inputs (which in other conditions represent waste) can be valuable assets for the production of bioenergy and biomaterials in biorefineries, contributing to the mitigation of environmental impact and the reduction of greenhouse gas production [6]. Some of the challenges of the bioeconomy are developing regulatory frameworks to guarantee biotechnological product safety, public investment in innovation, and stimuli to facilitate their insertion into the market [7]. It is also necessary that policies aimed at promoting the bioeconomy in the EU integrate an ample, balanced, and comprehensive vision of all the aspects that make up the framework of sustainable development [8]. In this regard, biorefineries are a fundamental element for advancing the bioeconomy since they provide value to waste and allow for circularity, as shown by the biorefining of apple pomace to produce valuable products, reduce waste, and promote sustainability [9, 10].

Due to its vast biodiversity and resources, Latin America has significant potential for moving toward a bioeconomy, and biorefining stands out among the potential areas for development. First, however, it is necessary to promote technological development, local solutions, social inclusion, and environmental awareness [11]. In Brazil, for example, there is an interest in biorefineries using elephant grass inputs to produce bioenergy, as well as other options to obtain butanol and acetone, such as sugar cane biorefineries, which are gaining acceptance globally due to the possibility of using these crops integrally [12–14]. In fact, since energy is a central issue and is linked to social and economic progress, lignocellulosic biorefinement represents an alternative for the production of biofuels and other processes aimed at obtaining value-added products in a global environment of energy crisis [15]; however, due to the nature of this type of biomass, pretreatment processes are required for its correct use, but it is increasingly accepted as a viable option to consolidate the circular bioeconomy and well-being [16, 17].

As a response to the concern of obtaining chemical products from biomass, first- and second-generation biofuels represent sustainable alternatives. Nevertheless, to make these alternatives feasible, it is necessary to rigorously evaluate them using methodologies such as life cycle assessment (LCA) or environmental impact and analyze possible adverse collateral effects to articulate realistic and workable solutions based on results [18]. This methodology can also be used to assess the sustainability of the types of biomass used in biorefineries; together with other parameters, and the use of decision-making analysis to improve environmental performance [19], to address the necessity of gradually replacing fossil fuels in the face of their imminent depletion, and as a way of mitigating global warming resulting from energy demands [20]. In cases such as manure processing, anaerobic digestion continues to be the best alternative for the production of renewable energy, but its numerous problems highlight the need to generate biorefining technology and implement comprehensive management schemes, especially in developing countries [21].

Although there are relevant studies concerned with biorefinery and its relationship with the bioeconomy that use bibliographic information for networks-based analysis, the value of this research lies in the integration of an updated and complete analysis including not only the study of this topic from a basic-science perspective, but also from a technical point of view. Derived from the above, the purpose of the present study was to identify and analyze trends around basic research (network analysis based on bibliographic data) and technological development related to biorefineries (analysis of patent documents) in connection with the principles of sustainability. This approach allowed, on the one hand, to map the main advances in this area of knowledge through the identification of different thematic clusters and relevant nodes of research and, on the other hand, to highlight the main technological sectors and other emerging sectors that will gain strength in the future, including the patent applicants and territories where this technology is to be commercialized. Thus, this methodology combines robust elements of a scientific and technological trajectory. The results of this study are relevant to understanding the industrial dynamics from research and development (R&D) on biorefineries and their relation with sustainability, and they show multiple topics of interest concerning the impact of this technology on society, the environment, and the economy.

## Methodological approach

Network analyses help to understand the dynamics and issues associated with specific areas of knowledge. Databases such as Web of Science (WoS) are used to carry out studies focused on different issues related to biorefineries and circular economy, although the need for additional research in the area is recognized, in addition to related topics such as life cycle assessment, process integration, and techno-economic analysis, as well as sustainability [22], including bibliometric studies on bio-waste [23]. Similarly, patent and scientific document analyses help to understand industry-academia collaboration mechanisms for joint development, for example, in the development of fuel cells [24]. Thus, patent analysis is relevant because it allows us to understand the value of these intangible assets from an intellectual, financial, and competition perspective since the integration of appropriate indicators for their analysis is essential; some of them are IPC classes and citations, among other indicators [25]. There are cases, such as the lignin biorefinery, in which the study of scientific production and patents has been useful to identify the increasing interest in this area of knowledge, which makes it possible to analyze the main technological advances, and identify relevant applicants [26] (Garlapati et al., 2020).

**Fig 1:**
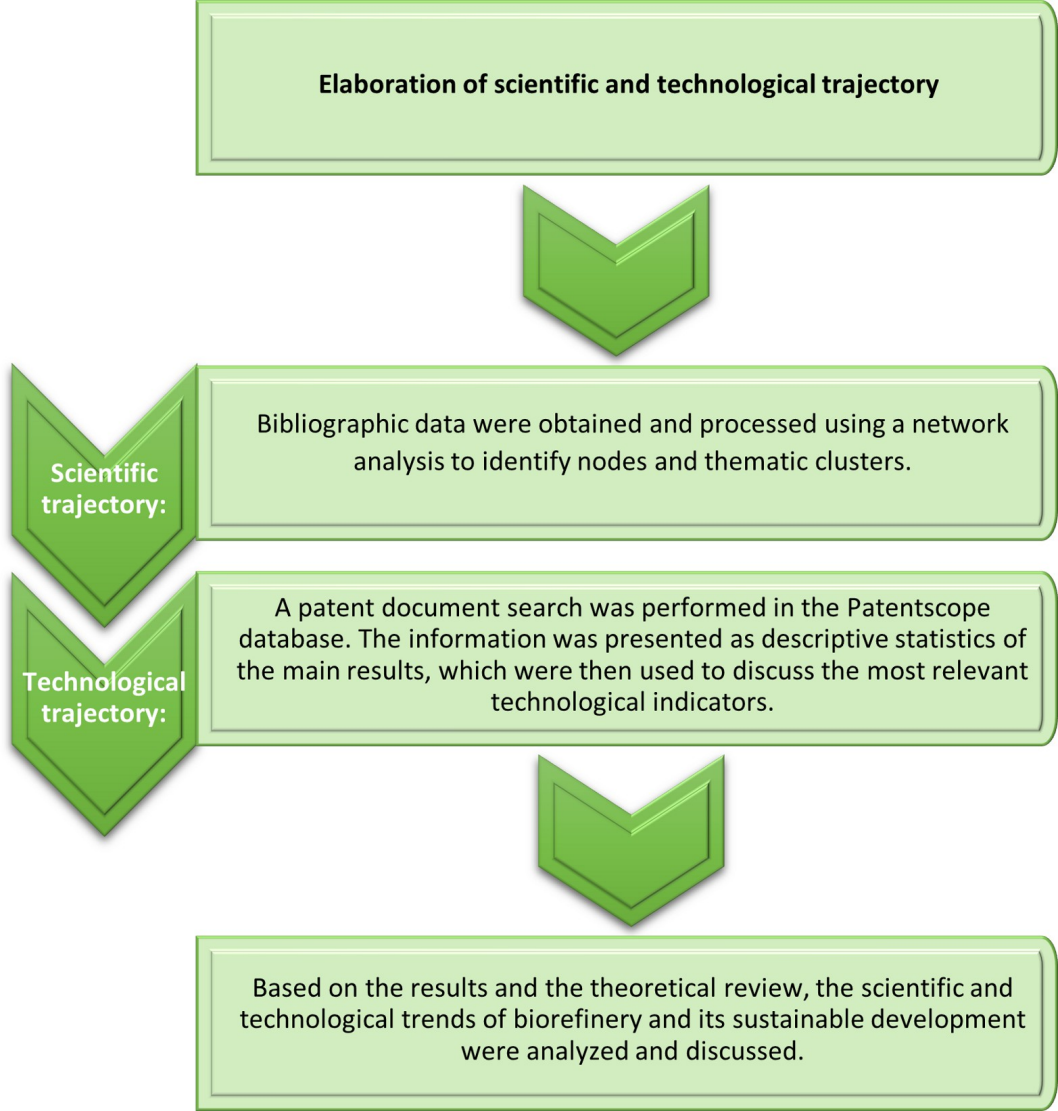
Methodological sequence. Source: Elaborated by the authors.

Thus, although there are related studies, this research addresses basic science and patents related to biorefineries and sustainable development in depth to answer the following question: What are the current trends of basic research and technological development around biorefineries and sustainable development? The methodological proposal consisted in carrying out a network (co-occurrence) analysis and a patent analysis and with two purposes: firstly, to identify the main thematic areas related to biorefineries and sustainability, and secondly, to pinpoint the main technological trends related to progress in this sector. Two searches were conducted for these purposes; the first aimed to identify academic documents in the Scopus database [27], which was selected due to its robustness and academic prestige, and the second search located patent documents in the Patentscope database (World Intellectual Property Organization, WIPO), which includes almost 100 million patent documents, including approximately 4 million PCT (Patent Cooperation Treaty) applications from a large number of intellectual property offices around the world [28] (see Fig 1).

### Network analysis based on bibliographic data

The first search was carried out in Scopus [29] using the following query in the fields: title, abstract, and keyword: (TITLE-ABS-KEY(biorefinery) OR TITLE-ABS-KEY (biorefineries) AND TITLE-ABS-KEY (sustainability). This search found 1,201 documents. These documents were 652 articles (54.3%), 236 reviews (19.7%), 143 book chapters (11.9%), 114 conference papers (9.5%), 18 books (1.5%), 12 conference reviews (1%), 8 editorials (0.7%), 6 notes (0.5%), 6 short Surveys (0.5%), 4 erratum (0.3%), 1 business article (0.1%), and 1 letter (0.1%). This bibliographic information was the basis for the elaboration of a co-occurrence analysis (for more information, see S1 Dataset) using VOSviewer software [30] and the following analysis criteria: 1.- Type of analysis: co-occurrence; 2.- Unit of analysis: Author keywords; 3.- Counting method: Full counting; 4.- Minimum number of occurrences of a word. 5. In this way, 2,819 keywords were identified, 149 of which had a frequency equal to or greater than five; these keywords were the basis for a network map created using the LinLog/Modularity normalization method [31], which identified topics of interest and their grouping within different thematic clusters related to biorefinery and sustainability.

### Analysis of patent documents

An advanced search was carried out in Patentscope [28] using the following criteria: 1.- Offices: All; 2.- Language: English; 3.- Automatic separation of words into lexemes was included; 4.- Patent documents were grouped to identify them as members of a simple patent family; and 5.- Literature other than that of patents was excluded from the search. As a result, the database produced 2,053 patent documents using the following search string: biorefinery OR biorefineries. This made it possible to integrate and analyze related information on countries or territories where applications were registered, as well as the main applicants, publication dates, and main IPC (International Patent Classification) categories, which served to identify the different technological areas where the most important advances in biorefinery are found.

One of the main limitations of this database is that its analysis bar shows a maximum of 50 results (items/groups) (for more information, see S1 Annex). Thus, for this study, only the twenty main results were considered in each category, except for patent documents ordered by publication date, in which case the entire period (1995–2020) was analyzed only through descriptive statistics because the data were very scattered. To cite a few examples, the standard deviation and the median for the number of documents per country of the main 20 cases were 270.1 and 3.5; in the case of documents per applicant, 24.3 and 18, and for documents by IPC code, 164.4 and 125.5, and the highest values for each of these items were 906, 106, and 761, respectively. The following section presents a brief discussion on the ten main patent documents according to the database’s search relevance criterion; this discussion exemplifies significant cases related to biorefinery development.

## Results analysis

**Fig 2:**
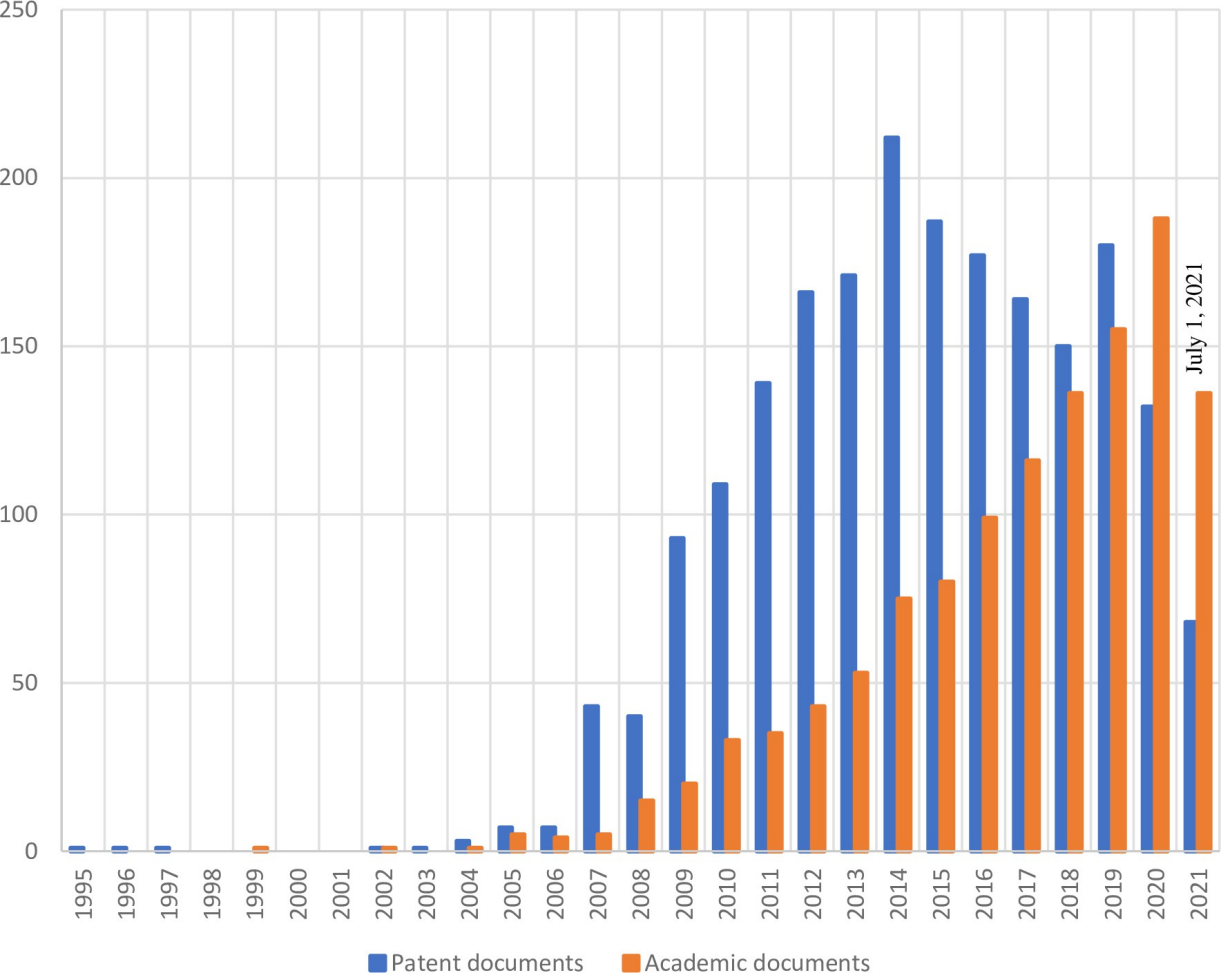
Academic and patent documents. Source: Prepared by the authors based on WIPO [28] & Scopus [29].

Fig 2 shows the evolution of scientific and patent document production; in both cases, we observed that production acquired a dynamic increase after 2005. However, academic document production grew steadily, as opposed to the case of patent document production, which peaked in 2014, and its growth was not sustained in the following years. Although this behavior might change in the future, it is possible for basic science production to continue increasing while the appearance of new technologies grows at a variable pace since many of these developments have not been able to be consolidated in the markets due to technical and economic feasibility issues.

The most relevant results for the network map are shown in this section. These are related to occurrences, links, and total link strength for each of the identified clusters, which highlight the most relevant themes and their connection with other topics of interest. In addition, in the analysis of patent documents, we present the territories where these inventions are protected, their applicants (often firms), their relevant technological sectors, and some examples of the most relevant patents in the field. All this information is important because it not only allowed us to determine the types of technology that have progressed the most but it also allowed for an approach to understanding market dynamics.

Based on the previously identified words, the co-occurrence analysis resulted in 149 nodes distributed in 10 clusters, with 1,658 links (L) and a total link strength (TLS) of 3,284 (for more information, see S2 Annex). The three most important nodes per cluster are listed below according to occurrence (O) (L/TLS/O): Cluster 1 (red): a) Sustainability assessment—30/43/22; b) Circular bioeconomy—30/43/18; and c) integrated biorefinery—24/32/17; Cluster 2 (green): a) Life cycle assessment—64/150/71; b) Microalgae—58/168/69; and c) Bioethanol—50/106/39. Cluster 3 (dark blue): a) Biofuel—71/158/52; b) Biodiesel 59/140/43; and c) Ethanol—43/99/30. Cluster 4 (yellow): a) Biorefinery—134/827/361; b) Circular economy—55/126/46; and c) Bioenergy—43/104/38. Cluster 5 (purple): a) Biofuels—73/216/75; b) Biorefineries—55/116/56; and c) Process design—23/38/15. Cluster 6 (light blue): a) Biomass—83/246/92; b) Renewable energy—31/46/16; c) Energy—34/48/14. Cluster 7 (orange): a) Fermentation—38/55/17; b) Food waste—25/43/14; and c) Enzymatic hydrolysis—20/28/13. Cluster 8 (brown): a) Pretreatment—41/95/34; b) Lignocellulosic biomass—39/87/32; and c) Lignin—36/62/26. Cluster 9 (fuchsia): a) Sustainability—123/512/224; b) Optimization—20/37/18; and c) Supply chain—12/22/13. Cluster 10 (pink): a) Lignocellulosic—16/20/6; and b) Logistics—11/8/5.

A comparison between the network analysis results and the theoretical and conceptual framework showed that the main nodes associated with these terms are related to biomass, microalgae, life cycle assessment, biofuels, techno-economic analysis, circular economy, and bioeconomy, among others. These items have numerous links with other terms, regardless of the size of the nodes. For example, the life cycle assessment node, with 64 links, is connected to concepts such as biorefinery, sustainability, biodiesel, ethanol, microalgae, and biomass, among others, as well as the biofuel node, with some of its 71 links associated with topics such as hydrogen, butanol, anaerobic digestion, green chemistry, sustainability evaluation, bioproducts, climate change, and many others.

**Fig 3:**
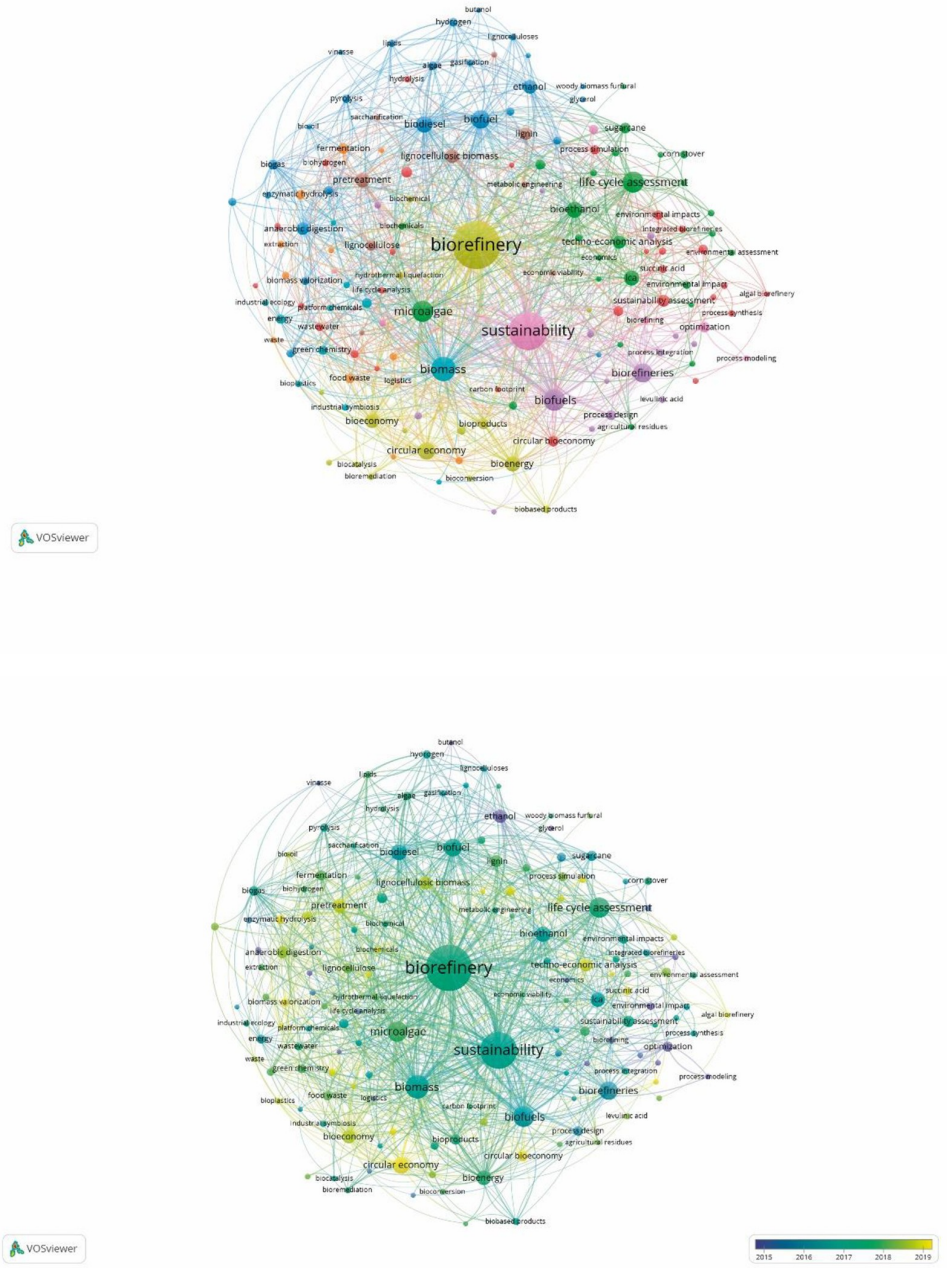
Co-occurrence analysis—keywords (author). Source: Authors’ elaboration based on Scopus [29, 30].

These results reveal a series of thematic clusters that can be grouped; for example, cluster 3 includes topics related to energy generation. Terms related to biofuel generation, as well as others mentioned above, such as life cycle assessment, are not only central themes around the concept of biorefinery: due to their many links, they are highly interactive with other elements, which reflects the dynamism in the area and highlights the interdisciplinary and multidisciplinary work that will foster new scientific and technological developments in the future, moving biorefining forward. As a consequence, the market will have to integrate strategies to favor the development of innovative and sustainable solutions into its business models. Fig 3 shows the network and the superimposed visualization. The first map shows the most important nodes, presented by number of occurrences, and the distance between nodes represents the degree of the relationships between terms. The second map presents the themes by publication year, highlighting the importance of the themes within a given period.

**Fig 4:**
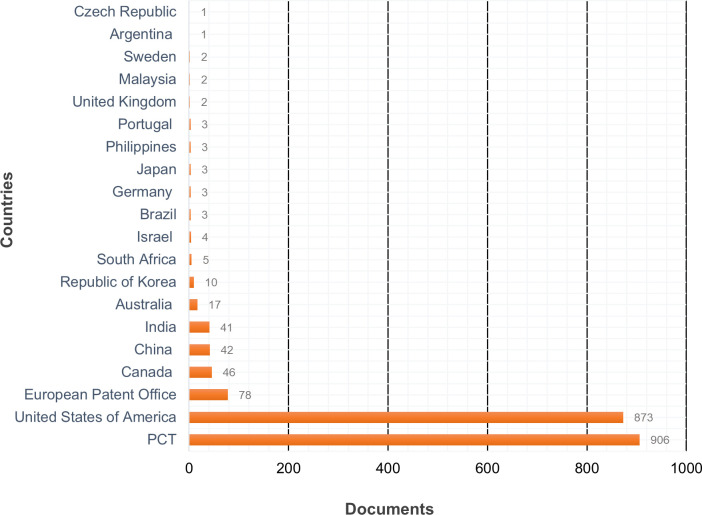
Countries with the highest number of patent documents. Source: Prepared by the authors based on WIPO [28].

Within the 20 most important results in the patent document analysis were the applications within the Patent Cooperation Treaty (PCT) (906), revealing an interest in protecting these inventions in territories other than where they were created to promote their broader commercialization. However, the United States has similar numbers (873), which is probably due to its importance as a technology market. The European Patent Office, Canada, China, India, and Australia also stand out with more modest results. Other European countries and some Asian countries like Japan and Korea can be found among the results; these are characterized by advances in other scientific and technological sectors. Developing countries such as South Africa and Brazil are beginning to emerge as regions of interest for this sector. Although sustainable development addresses a global concern, biorefining represents an especially advantageous alternative for countries that will require science and technology to face increasing environmental and social issues due to their large populations and expected industrial growth. By the same token, developing countries rich in biomass production ought to develop endogenous technologies that allow them to take advantage of these resources and thereby generate competitive advantages and solutions for their most pressing needs (see Fig 4).

**Fig 5:**
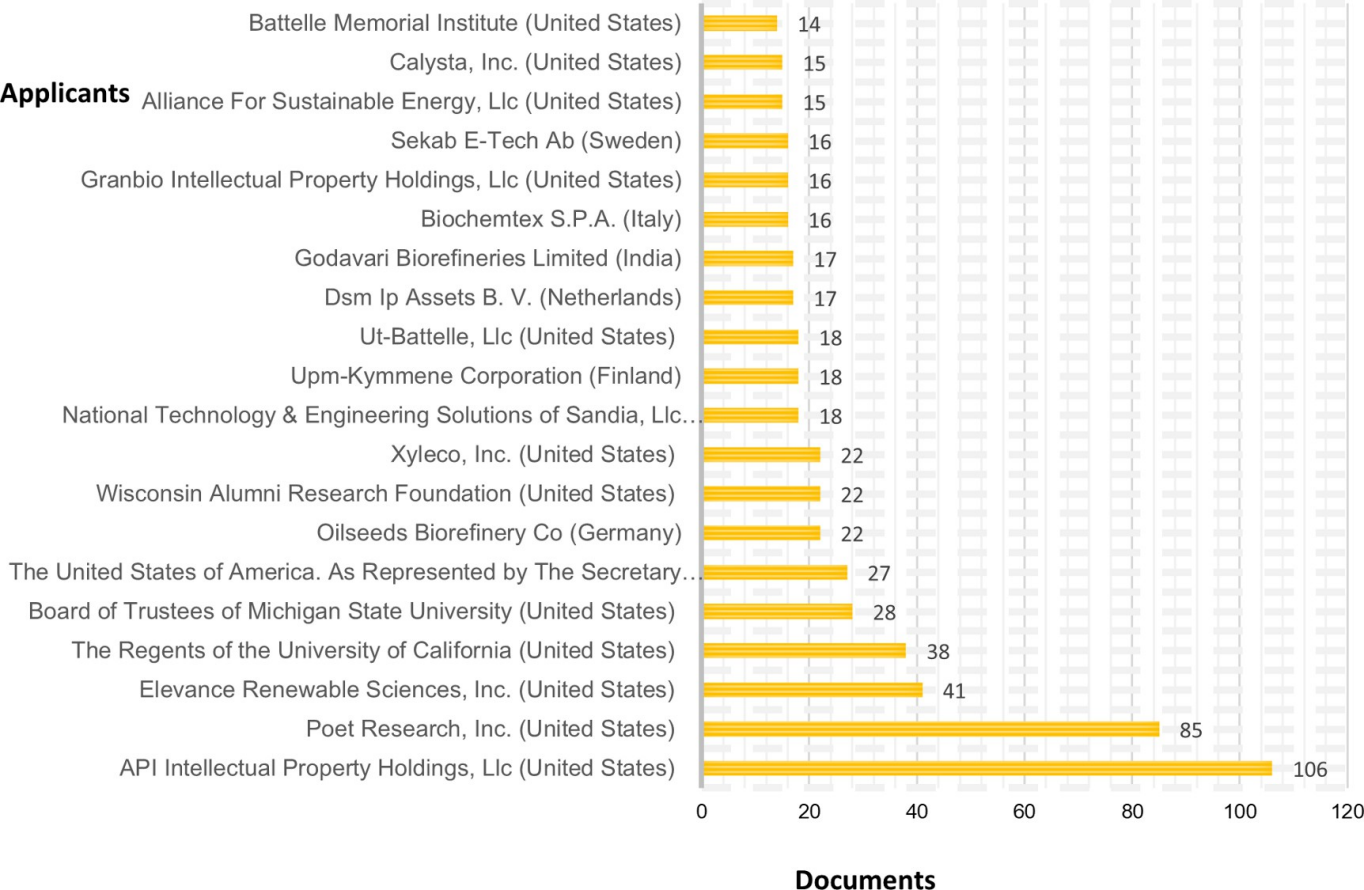
Top patent document applicants. Source: Prepared by the authors based on WIPO [28].

The role of the United States is supported by private companies, where the vast majority of applicants for these patent documents originate, as shown by the analysis of the 20 most important applicants. Likewise, after the United States, the role of European companies is relevant. In India, only Godavari Biorefineries Limited is included in the top 20 positions. Although other intellectual property offices’ databases from developing economies in different languages should be explored, the information from this analysis, based on the previously established criteria, reveals that companies mainly from developed economies are requesting the protection of technological developments related to bioreactors, highlighting the need to increase the participation of companies from developing countries, not only to generate local solutions but also to import technological applications and, thereby, obtain a technological income from these developments, although it will certainly be necessary to carefully plan these strategies to move forward in a steady but significant way (see Fig 5).

Thus, the three most important technological sectors are related to fermentation and enzymes that help to obtain chemical products, the use of microorganisms, and acyclic or carbocyclic compounds. Table 1 reveals a wide distribution of technological classes, reflecting the versatility of biorefining technology. Several related items can be observed, for example, polysaccharides and saccharides, as well as chemical and physical processes and technologies fundamental to developing biorefining devices for enzymological or microbiological applications and other technologies related to separation and wastewater treatment. The opportunities offered by biorefining are many and dynamic, although technical, economic, environmental, and social challenges should be prioritized for integrating a sustainable biorefinery. It is interesting to note that, despite the wide variety of technological classes offered by biorefining, traditional areas such as microbiology, fermentation processes, and the use of enzymes constitute one of the fundamental axes in the field, showing not only their current importance but also their evolution to identify new and improved applications.

**Table 1:** Main technological sectors, International Patent Classification (IPC).

No.	Code	Description	Documents
**1**	C12P	"Fermentation or enzyme-using processes to synthesise a desired chemical compound or composition or to separate optical isomers from a racemic mixture."	761
**2**	C12N	"Microorganisms or enzymes; compositions thereof; propagating, preserving, or maintaining microorganisms; mutation or genetic engineering; culture media…."	452
**3**	C07C	"Acyclic or carbocyclic compounds…"	281
**4**	D21C	"Production of cellulose by removing non-cellulose substances from cellulose-containing materials; regeneration of pulping liquors; apparatus therefor."	177
**5**	C08H	"Derivatives of natural macromolecular compounds (polysaccharides c08b; natural rubber C08C; natural resins or their derivatives C09F; working up pitch, asphalt or bitumen C10C 3/00)"	166
**6**	C10L	"Fuels not otherwise provided for; natural gas; synthetic natural gas obtained by processes not covered by subclasses C10G or C10K; liquefied petroleum gas; use of additives to fuels or fires; fire-lighters."	166
**7**	B01J	"Chemical or physical processes, e.g. catalysis or colloid chemistry; their relevant apparatus"	155
**8**	C10G	"Cracking hydrocarbon oils; production of liquid hydrocarbon mixtures, e.g. by destructive hydrogenation, oligomerisation, polymerisation (cracking to hydrogen or synthesis gas C01B; cracking or pyrolysis of hydrocarbon gases to individual hydrocarbons or mixtures thereof of definite or specified constitution C07C; cracking to cokes C10B); recovery of hydrocarbon oils from oil-shale, oil-sand, or gases; refining mixtures mainly consisting of hydrocarbons; reforming of naphtha; mineral waxes"	153
**9**	C08B	"Polysaccharides; derivatives thereof (polysaccharides containing less than six saccharide radicals attached to each other by glycosidic linkages C07H; fermentation or enzyme-using processes C12P 19/00; production of cellulose D21)"	138
**10**	C08L	"Compositions of macromolecular compounds (compositions based on polymerisable monomers C08F, C08G; artificial filaments or fibres D01F; textile treating compositions D06)"	133
**11**	C07D	"Heterocyclic compounds (macromolecular compounds C08)"	118
**12**	C13K	"Saccharides obtained from natural sources or by hydrolysis of naturally occurring disaccharides, oligosaccharides or polysaccharides (production of sucrose C13B; chemically synthesised sugars or sugar derivatives C07H; polysaccharides, e.g. starch, derivatives thereof C08B; malt C12C; fermentation or enzyme-using processes for preparing compounds containing saccharide radicals C12P 19/00)"	112
**13**	C12M	"Apparatus for enzymology or microbiology (installations for fermenting manure A01C 3/02; preservation of living parts of humans or animals A01N 1/02; brewing apparatus C12C; fermentation apparatus for wine C12G; apparatus for preparing vinegar C12J 1/10)"	105
**14**	C07G	"Compounds of unknown constitution (sulfonated fats, oils or waxes of undetermined constitution C07C 309/62)"	102
**15**	B01D	"Separation (separating solids from solids by wet methods B03B, B03D, by pneumatic jigs or tables B03B, by other dry methods B07; magnetic or electrostatic separation of solid materials from solid materials or fluids, separation by high-voltage electric fields B03C; centrifuges B04B; vortex apparatus B04C; presses per se for squeezing-out liquid from liquid-containing material B30B 9/02)"	91
**16**	C11B	"Producing, e.g. by pressing raw materials or by extraction from waste materials, refining or preserving fats, fatty substances, e.g. lanolin, fatty oils or waxes; essential oils; perfumes (drying-oils C09F)"	90
**17**	C01B	"Non-metallic elements; compounds thereof (fermentation or enzyme-using processes for the preparation of elements or inorganic compounds except carbon dioxide C12P 3/00; production of non-metallic elements or inorganic compounds by electrolysis or electrophoresis C25B)"	74
**18**	C02F	"Treatment of water, waste water, sewage, or sludge (processes for making harmful chemical substances harmless, or less harmful, by effecting a chemical change in the substances A62D 3/00; separation, settling tanks or filter devices B01D; special arrangements on waterborne vessels of installations for treating water, waste water or sewage, e.g. for producing fresh water, B63J; adding materials to water to prevent corrosion C23F; treating radioactively-contaminated liquids G21F 9/04)"	68
**19**	C08G	"Macromolecular compounds obtained otherwise than by reactions only involving carbon-to-carbon unsaturated bonds (fermentation or enzyme-using processes to synthesise a desired chemical compound or composition or to separate optical isomers from a racemic mixture C12P)"	68
**20**	C12R	"Indexing scheme associated with subclasses C12C-C12Q, relating to microorganisms"	59

**Source:** Prepared by the authors based on WIPO [28, 32].

When sorting patent documents by search relevance, the first ten documents to be displayed were registered in the United States, the European Patent Office, and Canada. However, half of these ten documents are PCT applications. Thus, as previously mentioned, there is a clear intention to bring these technologies to other markets regardless of the original place of registration, revealing that these types of inventions have a more significant global potential for industrial and commercial use. The listed technologies are related to biorefinery control systems, integrated and sustainable biorefinery, methods for converting traditional plants into biorefineries, biorefinery systems, submerged biorefinery chamber, algae biorefinery, lignocellulosic biorefinery, and applications related to bioproducts, biofuels, and enzymes. It is thus clear, as these examples show, that in searching for sustainable solutions, biorefinery-related systems are being chosen over traditional refining systems; in this context, it will be necessary to generate different disciplinary and transdisciplinary solutions in addition to alternatives using raw materials as abundant as lignocellulose (see Table 2).

**Table 2:** Top ten patents by search relevance.

No.	Title	Office	Publication number	IPC
**1**	“Biorefinery control system, components therefor, and methods of use.”	United States of America	20140030695	C12M 1/36; C12Q 3/00
**2**	“Integrated biorefinery.”	United States of America	20160348134	C12P 7/14; C12M 3/00; C12M 1/26
**3**	“Sustainable biorefinery.”	PCT	WO/2014/072580	C12P 7/56; C12P 7/08; C12P 7/06; A23K 1/00; C12R 1/225
**4**	“Method for converting a conventional oil, petrochemical, or chemical plant into a biorefinery.”	European Patent Office	3450524	C10G 7/00; C10G 1/06; C10G 3/00; C10G 7/06
**5**	“Biorefinery control system, components therefor, and methods of use.”	United States of America	20190078049	C12M 1/36; C12M 1/00; C12M 1/08; C12Q 3/00; C12M 1/107
**6**	“Biorefinery system, components therefor, methods of use, and products derived therefrom.”	PCT	WO/2012/100093	B09B 3/00; C02F 11/04; C12M 3/00; C12P 1/00; C12R 1/89
**7**	“Submerged biorefinery chamber for large scale tailings ponds.”	Canada	3024256	C02F 3/00; B03B 9/02; C02F 1/44; C02F 3/32; C12M 1/00; C12P 1/00; C02F 3/34
**8**	“Biorefinery and method for revamping a conventional refinery of mineral oils into said biorefinery.”	PCT	WO/2015/181279	C10G 3/00
**9**	“Microalgae biorefinery for biofuel and valuable products production.”	PCT	WO/2015/044721	C10L 1/02; C10L 3/08; C12M 1/00
**10**	“Enzyme recovery sorbent, enzyme recovery unit, lignocellulosic biorefinery, process for recycling enzymes, and renewable material.”	PCT	WO/2010/129101	C12N 9/42; C12P 7/10; C07K 1/16; C07K 1/22

**Source:** Prepared by the authors based on WIPO [28].

### Biorefinery and sustainable development

Due to its numerous applications in different areas, the biotechnology industry represents a significant source of economic benefits. In particular, biotechnology will play a decisive role in the chemistry and energy sectors as a pathway for generating environmentally friendly solutions [33]. For example, food waste is a valuable input for biorefineries, and the use of bioprocesses (methanogenesis and fermentation, among others) offers the possibility of obtaining value-added products such as biofuels, biopolymers, and chemical substances, to name a few [34]. Thus, recognizing the value of biowaste is essential for the development of processes aimed at obtaining biofuels and other products of interest [35]. Using organic waste processed by biorefinery under a cost-benefit strategy can yield competitive results in obtaining H_2_ and other bio-products and improving the social acceptance of this type of technology [36]. Nevertheless, biorefining should be carried out using robust and standardized guidelines based on life cycle assessment (LCA) criteria to be efficient in environmental, production, and economic terms [37].

Another valuable input for biorefining processes is fish waste; through anaerobic co-fermentation processes, this waste can be transformed into different products, including methane, although its economic feasibility may be conditioned by its production yield and market selling costs, which requires public policies to subsidize industrial operations of this kind [38]. The many sources of coffee waste available for the production of energy and biological products are yet another area of opportunity for applications involving integrated- and cascade-type biorefineries; these strategies require more research and development (R&D), as well as regulatory frameworks, investment, indicators, and other elements to be implemented and contribute to the circular bioeconomy [39]. On the other hand, despite the abundantly available biomass, the cellulosic biorefinery has important challenges in terms of technological development and operating costs to achieve market-ready biomaterials, biofuels, and other competitive derived products [40].

As a source of value-added products, biorefining requires collaboration among different actors, such as the industrial and academic sectors, and support for R&D and innovation. Thus, in certain regions, the forest biorefinery is deemed a central element of a competitive bioeconomy since it has applications relevant to the market, and the technological progress around the use of woody biomass continues to be developed [41, 42]. In general, biorefining offers a fair number of solutions based on the use of biomass to obtain chemical products and other materials, and they can be used to produce energy [43]. Some examples are succinic acid obtained from citrus peel [44] and the use of switchgrass (lignocellulosic biomass) to obtain energy, bioethanol, and other chemicals [45]. In fact, lignin biorefining has significant potential for the generation of value-added products, including different chemicals, pharmaceutical ingredients, polymers, and fuels, which contribute to production chain performance [46], but despite its progress in terms of profitability and sustainability, there are several technical problems to address in order to make it efficient [47].

The transition from traditional biorefining to waste-based biorefining is an interesting case, involving different economic opportunities and technical challenges [48]; among these are the integration of separation technologies to increase the competitiveness of production processes [49]; biorefinery, which uses dairy waste to produce energy and obtain bioplastics and chemicals [50], and membrane-based processes in biorefineries [51]. An interesting case in this category is algae and microalgae refinery, which offer many alternatives for energy production and non-energy products [52–55], for example, microalgae biodiesel, which is environmentally friendly, and its cultivation contributes to CO_2_ capture [56]. In addition, the microalgae biorefinery can benefit from tools such as the Internet of Things (IoT) to optimize different processes necessary for its operation, although its role in the sustainable bioeconomy requires financial support to be gradually positioned [57, 58].

**Fig 6:**
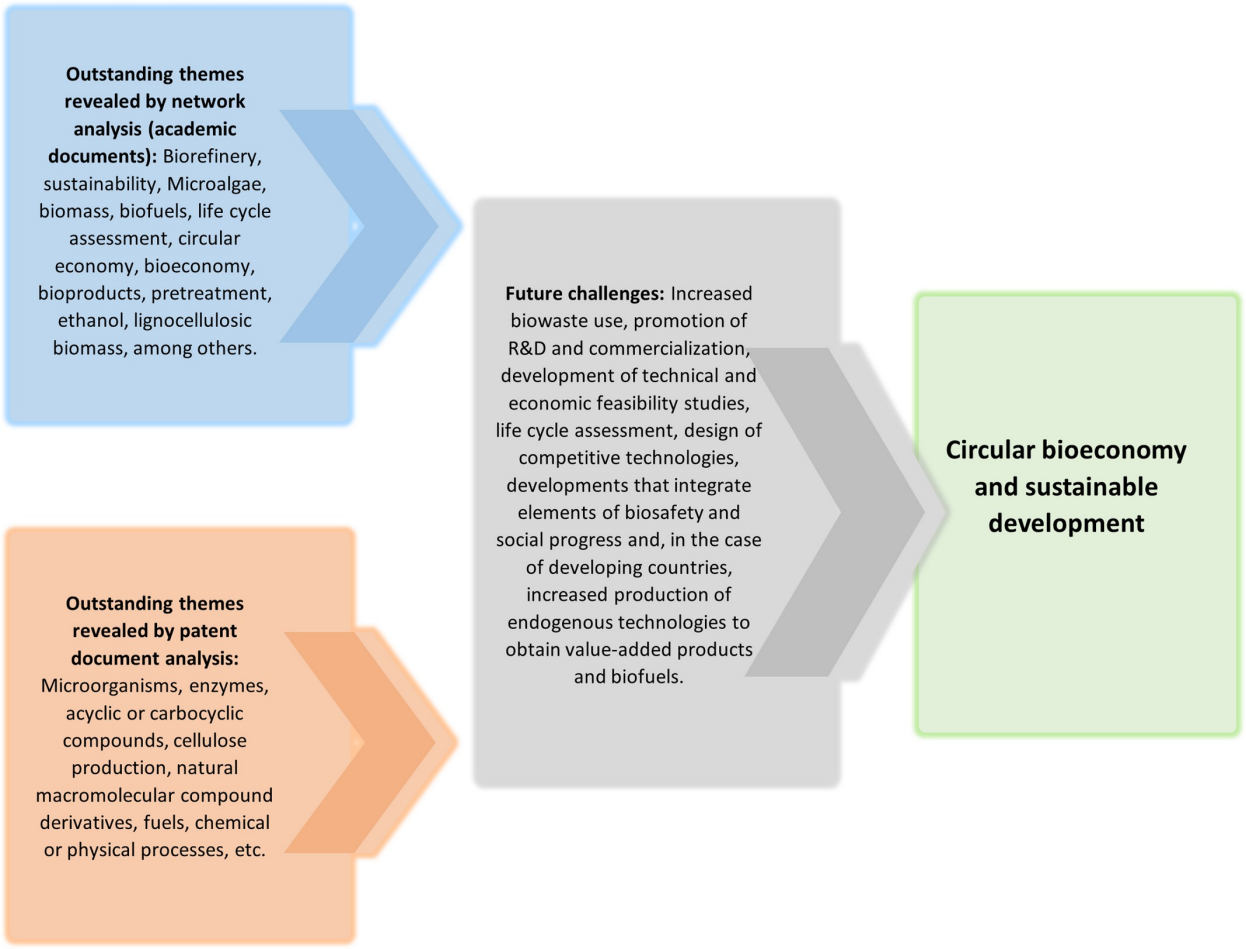
Biorefining, bioproducts, and biofuels. Source: Prepared by the authors.

Consequently, the most significant challenge in designing and configuring sustainable biorefineries is product manufacturing and the integration of complementary processes [59, 60]. Greenhouse gases (GHG) are still a growing concern, so environmentally friendly technologies and renewable energy sources will require policies including adequate incentives for their development [61]. Thus, biorefining can be seen as a mechanism for promoting the bioeconomy and reducing our dependence on petroleum under a framework of actions to achieve sustainability. However, there are challenges to be overcome, such as increasing market penetration, operationalizing the technical and economic viability of the processes, and increasing efficiency and investment in R&D [62]. Fig 6 presents the most relevant themes resulting from the network study based on bibliographic data and the patent document analysis, which were also addressed during the theoretical discussion, in addition to the future challenges of biorefining for the achievement of a circular bioeconomy and sustainable development.

## Conclusions

At present, there is a strong interest in seeking alternatives to non-renewable resources. In this context, the different types of biomass sources represent an opportunity since they can be transformed into valuable products and energy sources. Biorefining constitutes a central element in this process. The goal is to generate a sustainable framework for industrial development, increased competitiveness, and economic and social progress. However, it is essential to continue working on regulatory frameworks and biosafety issues, promoting R&D in the sector, and providing the necessary stimuli to enable the implementation of this type of technology. However, for this group of emerging technological developments, technical and economic feasibility represent significant challenges, including confronting the current dominance of traditional technologies, such as those integrated into the conventional refinery.

Although this group of technologies also seeks to contribute to sustainable development, it is necessary to carry out environmental impact evaluations to prevent adverse effects that could take place during development, implementation, and useful life to support adequate decision-making. The significant scientific and technological supply surrounding biorefining will allow for the advancement of biorefining in technical, economic, environmental, and social fields; thus, the development of indicators will play a fundamental role in its constant monitoring and evaluation. The present study highlights the current basic research and technological development trends in the field of biorefining and sustainability, as well as its challenges and progress, to answer the research question initially raised.

The network analysis on biorefining and sustainability revealed the relevance of topics such as biomass, biofuels, bioeconomy, circular economy, and life cycle assessment, among many others that we have discussed throughout this research. Conversely, the analysis of patent documents revealed a significant number of technological classes associated with products and many types of inputs necessary for the economic activity, as well as the technologies needed to develop biorefining further and the role that microbiology and enzymes will continue to play will be fundamental. The proposed methodology represents a comprehensive approach to the study of both the scientific and technological trajectories. This study shows existing trends in the basic research and technological development of biorefining in combination with sustainability and maps the main themes addressed by this area of knowledge.

Although this topic has been studied from a basic-research perspective and using bibliographic information and networks analysis, the lack of patent studies in the area has been identified. Thus, this study provides an integral and updated vision of the scientific and technological panorama of biorefinery and its contribution to sustainability through the production of energy and value-added products. It represents a starting point for the definition and formulation of future studies. Undoubtedly, biomass is the fundamental input of biorefinery, and the network map shows its many possibilities for exploitation. Traditional technologies such as fermentation and the use of enzymes and microorganisms will remain as the most crucial axis of this area of knowledge due to their nature. However, it will be necessary to develop alternative technologies that complement the former and contribute to the achievement of the technical and economic viability of this type of projects.

Only through these actions will it be possible to develop the circular bioeconomy and, with it, progress toward the construction of sustainable development strategies considering the life cycle and environmental impact of products and generating sustainable energy sources. The dynamics of the biorefinery are in constant and rapid change, and this study shows that the most recent topics addressed have to do with economic themes, as well as technical ones, such as lignocellulosic biomass, pretreatment, and enzymatic hydrolysis, which highlight the challenges facing the biorefinery. However, one of the main limitations of this study is the lack of discussion on specific cases; therefore, selected cases will need to be investigated, and other sources of information will have to be explored in the future. The results of this study reveal that the accumulated scientific and technological progress of biorefining is abundant.

## Supporting information

S1 Dataset Database for the network. (CSV)

S1 Annex Annex I. Patent document indicators.
(DOCX)

S2 Annex Annex II. Co-occurrence analysis.
(DOCX)

S1 Graphical abstract (TIF)
