# Supplementary material for: Biorefinery and sustainability for the production of biofuels and value-added products: A trends analysis based on network and patent analysis
Source: PLoS One. 2023 Jan 12;18(1):e0279659. doi: 10.1371/journal.pone.0279659 (PMC9836267; doi:10.1371/journal.pone.0279659)
Supplement: S1 Annex — Patent document indicators. (DOCX) [file pone.0279659.s002.docx]

**Annex II. Patent document indicators**

| No. | Countries | Documents | Applicants | Documents | IPC code | Documents | Publication dates | Documents |
| --- | --- | --- | --- | --- | --- | --- | --- | --- |
| 1 | PCT | 906 | API Intellectual Property Holdings, Llc | 106 | C12P | 761 | 1995 | 1 |
| 2 | United States of America | 873 | Poet Research, Inc. | 85 | C12N | 452 | 1996 | 1 |
| 3 | European Patent Office | 78 | Elevance Renewable Sciences, Inc. | 41 | C07C | 281 | 1997 | 1 |
| 4 | Canada | 46 | The Regents of the University of California | 38 | D21C | 177 | 1998 | 0 |
| 5 | China | 42 | Board of Trustees of Michigan State University | 28 | C08H | 166 | 1999 | 0 |
| 6 | India | 41 | The United States of America. As Represented by The Secretary of Agriculture | 27 | C10L | 166 | 2000 | 0 |
| 7 | Australia | 17 | Oilseeds Biorefinery Co | 22 | B01J | 155 | 2001 | 0 |
| 8 | Republic of Korea | 10 | Wisconsin Alumni Research Foundation | 22 | C10G | 153 | 2002 | 1 |
| 9 | South Africa | 5 | Xyleco, Inc. | 22 | C08B | 138 | 2003 | 1 |
| 10 | Israel | 4 | National Technology & Engineering Solutions of Sandia, Llc | 18 | C08L | 133 | 2004 | 3 |
| 11 | Brazil | 3 | Upm-Kymmene Corporation | 18 | C07D | 118 | 2005 | 7 |
| 12 | Germany | 3 | Ut-Battelle, Llc | 18 | C13K | 112 | 2006 | 7 |
| 13 | Japan | 3 | Dsm Ip Assets B. V. | 17 | C12M | 105 | 2007 | 43 |
| 14 | Philippines | 3 | Bodavari Biorefineries Limited | 17 | C07G | 102 | 2008 | 40 |
| 15 | Portugal | 3 | Biochemtex S.P.A. | 16 | B01D | 91 | 2009 | 93 |
| 16 | United Kingdom | 2 | Granbio Intellectual Property Holdings, Llc | 16 | C11B | 90 | 2010 | 109 |
| 17 | Malaysia | 2 | Sekab E-Tech Ab | 16 | C01B | 74 | 2011 | 139 |
| 18 | Sweden | 2 | Alliance For Sustainable Energy, Llc | 15 | C02F | 68 | 2012 | 166 |
| 19 | Argentina | 1 | Calysta, Inc. | 15 | C08G | 68 | 2013 | 171 |
| 20 | Czech Republic | 1 | Battelle Memorial Institute | 14 | C12R | 59 | 2014 | 212 |
| 21 | Denmark | 1 | Pioneer Hi Bred International Inc. | 14 | C11C | 55 | 2015 | 187 |
| 22 | Spain | 1 | Annikki GmbH | 13 | C07H | 54 | 2016 | 177 |
| 23 | Finland | 1 | Council of Scientific and Industrial Research | 13 | C07K | 53 | 2017 | 164 |
| 24 | Indonesia | 1 | Lignol Innovations Ltd. | 13 | C08J | 50 | 2018 | 150 |
| 25 | Mexico | 1 | Metabolix, Inc. | 13 | D21H | 46 | 2019 | 180 |
| 26 | New Zealand | 1 | Basf Se | 12 | C10J | 41 | 2020 | 132 |
| 27 | Romania | 1 | Berlin, Alex | 11 | C12Q | 39 | 2021 | 68 |
| 28 | Russian Federation | 1 | Exxonmobil Chemicel Patents Inc. | 11 | A61K | 38 |  |  |
| 29 |  |  | Narendranath Neelakantam V | 11 | A23K | 34 |  |  |
| 30 |  |  | Virginia Tech Intellectual Properties Inc. | 11 | A23L | 33 |  |  |
| 31 |  |  | Archer Daniels Midland Company | 10 | C10B | 33 |  |  |
| 32 |  |  | Charlson David Charles | 10 | G01N | 33 |  |  |
| 33 |  |  | Forest Concepts, Llc. | 10 | D21B | 31 |  |  |
| 34 |  |  | Honda Motor Ltd. | 10 | C09K | 27 |  |  |
| 35 |  |  | Inbicon A/S | 10 | C08K | 26 |  |  |
| 36 |  |  | Roal Oy | 10 | H01M | 26 |  |  |
| 37 |  |  | Sandia Co. | 10 | C08F | 24 |  |  |
| 38 |  |  | Sundrop Fuels Inc. | 10 | C09D | 23 |  |  |
| 39 |  |  | Carbon Tech Holdings Llc. | 9 | C09J | 23 |  |  |
| 40 |  |  | Cmblu Projekt Ag | 9 | A01H | 21 |  |  |
| 41 |  |  | Cornell University | 9 | A01N | 21 |  |  |
| 42 |  |  | Danisco Us Inc. | 9 | B82Y | 21 |  |  |
| 43 |  |  | Fpinnovations | 9 | C11D | 20 |  |  |
| 44 |  |  | Raindance Tech Inc. | 9 | C04B | 15 |  |  |
| 45 |  |  | Rennovia, Inc. | 9 | D01F | 15 |  |  |
| 46 |  |  | Teknologian Tutkimuskeskus Vtt Oy | 9 | A01G | 14 |  |  |
| 47 |  |  | The Research Foundation for the State University of New York | 9 | B09B | 14 |  |  |
| 48 |  |  | Genomatica, Inc. | 8 | C05F | 14 |  |  |
| 49 |  |  | Iowa State University Research Foundation Inc. | 8 | C25B | 14 |  |  |
| 50 |  |  | Korea Research Institute of Chemical Technology | 8 | B01F | 13 |  |  |

**Source:** Prepared by the authors based on WIPO (2021 a).
