# Supplementary material for: Biorefinery and sustainability for the production of biofuels and value-added products: A trends analysis based on network and patent analysis
Source: PLoS One. 2023 Jan 12;18(1):e0279659. doi: 10.1371/journal.pone.0279659 (PMC9836267; doi:10.1371/journal.pone.0279659)
Supplement: S2 Annex — Co-occurrence analysis. (DOCX) [file pone.0279659.s003.docx]

**Annex II. Co-occurrence analysis**

| **Cluster 1** |  |  |  | **Cluster 2** |  |  |  | **Cluster 3** |  |  |  | **Cluster 4** |  |  |  |
| --- | --- | --- | --- | --- | --- | --- | --- | --- | --- | --- | --- | --- | --- | --- | --- |
|  |  |  |  |  |  |  |  |  |  |  |  |  |  |  |  |
| items |  |  |  | items |  |  |  | items |  |  |  | items |  |  |  |
|  | links | Total link strength | occurrences |  | links | Total link strength | occurrences |  | links | Total link stregth | occurrences |  | links | Total link stregth | occurrences |
| adsorption | 9 | 10 | 6 | agricultural residues | 10 | 16 | 6 | algae | 22 | 47 | 12 | biobased products | 13 | 24 | 9 |
| algal biorefinery | 11 | 12 | 5 | allocation | 12 | 17 | 8 | anaerobic digestion | 46 | 78 | 26 | biocatalysis | 7 | 11 | 6 |
| aquaculture | 8 | 10 | 6 | biobased economy | 9 | 12 | 5 | bio-oil | 18 | 25 | 5 | biochemical | 16 | 20 | 7 |
| bioactive compounds | 20 | 31 | 10 | biochemicals | 24 | 32 | 8 | bio-refinery | 16 | 21 | 10 | bioconversion | 7 | 11 | 6 |
| biohydrogen | 16 | 19 | 7 | bioethanol | 50 | 106 | 39 | biochar | 25 | 35 | 12 | bioeconomy | 50 | 105 | 32 |
| carbon footprint | 11 | 11 | 5 | brazil | 10 | 12 | 5 | biodiesel | 59 | 140 | 43 | bioenergy | 43 | 104 | 38 |
| circular bioeconomy | 30 | 43 | 18 | cellulosic ethanol | 16 | 22 | 9 | biofuel | 71 | 158 | 52 | bioproducts | 30 | 60 | 18 |
| cyanobacteria | 10 | 10 | 5 | climate change | 24 | 35 | 11 | biogas | 35 | 61 | 15 | biorefinery | 134 | 827 | 361 |
| economic analysis | 13 | 13 | 6 | corn stover | 19 | 20 | 10 | biomass valorization | 19 | 26 | 11 | bioremediation | 16 | 22 | 7 |
| environmental impacts | 22 | 36 | 13 | eco-efficiency | 15 | 25 | 8 | biorefinery design | 12 | 14 | 10 | biotechnology | 19 | 19 | 5 |
| fatty acids | 13 | 15 | 6 | economic assessment | 9 | 15 | 6 | butanol | 11 | 15 | 5 | circular economy | 55 | 126 | 46 |
| hydrolysis | 14 | 19 | 7 | economics | 11 | 13 | 6 | ethanol | 43 | 99 | 30 | enzymes | 5 | 10 | 6 |
| integrated biorefineries | 13 | 18 | 9 | environmental assessment | 13 | 16 | 7 | gasification | 25 | 31 | 8 | greenhouse gas emissions | 10 | 11 | 5 |
| integrated biorefinery | 24 | 32 | 17 | environmental impact | 20 | 28 | 11 | glycerol | 9 | 9 | 6 | hydrothermal liquefaction | 17 | 23 | 9 |
| life cycle analysis | 11 | 12 | 7 | environmental sustainability | 20 | 29 | 15 | hydrogen | 26 | 45 | 12 | sustainable agriculture | 12 | 15 | 5 |
| life cycle assessment (lca) | 29 | 44 | 16 | furfural | 12 | 12 | 5 | life cycle sustainability assessment | 7 | 10 | 5 | wastewater treatment | 11 | 21 | 10 |
| life-cycle assessment | 12 | 12 | 6 | green biorefinery | 8 | 9 | 6 | lignocelluloses | 18 | 38 | 7 |  |  |  |  |
| multi-objective optimization | 9 | 14 | 9 | lactic acid | 18 | 20 | 8 | lipids | 23 | 43 | 9 |  |  |  |  |
| polyhydroxyalkanoates | 8 | 10 | 5 | lca | 35 | 74 | 37 | pyrolysis | 37 | 59 | 12 |  |  |  |  |
| process simulation | 20 | 38 | 14 | life cycle assessment | 64 | 150 | 71 | sustainable biorefinery | 12 | 18 | 11 |  |  |  |  |
| process synthesis | 13 | 18 | 8 | microalgae | 58 | 168 | 69 | syngas | 27 | 40 | 7 |  |  |  |  |
| sensitivity analysis | 13 | 17 | 5 | sugarcane | 32 | 59 | 19 | thermochemical conversion | 13 | 14 | 5 |  |  |  |  |
| succinic acid | 15 | 19 | 10 | switchgrass | 8 | 11 | 6 | vinasse | 15 | 20 | 5 |  |  |  |  |
| sustainability analysis | 24 | 35 | 14 | techno-economic analysis | 40 | 68 | 28 | waste management | 22 | 38 | 11 |  |  |  |  |
| sustainability assessment | 30 | 43 | 22 | techno-economic assessment | 15 | 19 | 7 | woody biomass | 11 | 13 | 5 |  |  |  |  |
| technoeconomic analysis | 9 | 9 | 5 | uncertainty | 17 | 19 | 9 |  |  |  |  |  |  |  |  |
| uncertainty analysis | 19 | 23 | 8 |  |  |  |  |  |  |  |  |  |  |  |  |
| wastewater | 20 | 32 | 11 |  |  |  |  |  |  |  |  |  |  |  |  |
|  |  |  |  |  |  |  |  |  |  |  |  |  |  |  |  |
|  |  |  |  |  |  |  |  |  |  |  |  |  |  |  |  |
| **Cluster 5** |  |  |  | **Cluster 6** |  |  |  | **Cluster 7** |  |  |  | **Cluster 8** |  |  |  |
|  |  |  |  |  |  |  |  |  |  |  |  |  |  |  |  |
| items |  |  |  | items |  |  |  | items |  |  |  | items |  |  |  |
|  | links | Total link strength | occurrences |  | links | Total link strength | occurrences |  | links | Total link stregth | occurrences |  | links | Total link stregth | occurrences |
| biofuels | 73 | 216 | 75 | biomass | 83 | 246 | 92 | bio-based products | 22 | 31 | 9 | cellulose | 13 | 22 | 6 |
| biomass conversion | 13 | 18 | 7 | bioplastics | 21 | 24 | 7 | bioprocessing | 13 | 16 | 6 | economic viability | 9 | 12 | 5 |
| biomaterials | 15 | 21 | 9 | catalysis | 15 | 21 | 8 | chemicals | 12 | 17 | 5 | lignin | 36 | 62 | 26 |
| biopolymers | 22 | 25 | 9 | chp | 23 | 28 | 5 | enzymatic hydrolysis | 20 | 28 | 13 | lignocellulose | 37 | 60 | 17 |
| biorefineries | 55 | 116 | 56 | energy | 34 | 48 | 14 | extraction | 17 | 25 | 9 | lignocellulosic biomass | 39 | 87 | 32 |
| food security | 9 | 10 | 5 | green chemistry | 27 | 44 | 14 | fermentation | 38 | 55 | 17 | metabolic engineering | 13 | 17 | 7 |
| hydrochar | 10 | 12 | 5 | industrial ecology | 13 | 14 | 7 | food waste | 25 | 43 | 14 | pretreatment | 41 | 95 | 34 |
| levulinic acid | 16 | 18 | 8 | industrial symbiosis | 16 | 20 | 8 | transesterification | 12 | 18 | 5 | renewable resources | 17 | 21 | 5 |
| macroalgae | 11 | 17 | 9 | platform chemicals | 23 | 32 | 8 | valorization | 11 | 16 | 9 | saccharification | 18 | 23 | 5 |
| optimisation | 6 | 8 | 6 | renewable energy | 31 | 46 | 16 | value-added products | 17 | 24 | 9 |  |  |  |  |
| process design | 23 | 38 | 15 | sustainable chemistry | 9 | 11 | 5 | waste | 18 | 25 | 6 |  |  |  |  |
| process integration | 22 | 29 | 9 |  |  |  |  |  |  |  |  |  |  |  |  |
| process intensification | 7 | 9 | 6 |  |  |  |  |  |  |  |  |  |  |  |  |
| sustainable development | 11 | 12 | 6 |  |  |  |  |  |  |  |  |  |  |  |  |
| waste biorefinery | 13 | 14 | 5 |  |  |  |  |  |  |  |  |  |  |  |  |
|  |  |  |  |  |  |  |  |  |  |  |  |  |  |  |  |
|  |  |  |  |  |  |  |  |  |  |  |  |  |  |  |  |
| **Cluster 9** |  |  |  | **Cluster 10** |  |  |  |  |  |  |  |  |  |  |  |
|  |  |  |  |  |  |  |  |  |  |  |  |  |  |  |  |
| items |  |  |  | items |  |  |  |  |  |  |  |  |  |  |  |
|  | links | Total link strength | occurrences |  | links | Total link strength | occurrences |  |  |  |  |  |  |  |  |
| biorefining | 11 | 21 | 8 | lignocellulosic | 16 | 20 | 6 |  |  |  |  |  |  |  |  |
| fractionation | 9 | 20 | 7 | logistics | 8 | 11 | 5 |  |  |  |  |  |  |  |  |
| optimization | 20 | 37 | 18 |  |  |  |  |  |  |  |  |  |  |  |  |
| process modeling | 6 | 13 | 6 |  |  |  |  |  |  |  |  |  |  |  |  |
| supply chain | 12 | 22 | 13 |  |  |  |  |  |  |  |  |  |  |  |  |
| sustainability | 123 | 512 | 224 |  |  |  |  |  |  |  |  |  |  |  |  |

**Source:** Prepared by the authors based on Scopus (2021 b) and VOSviewer (2021).
