# Supplementary figures and images for: Biorefinery and sustainability for the production of biofuels and value-added products: A trends analysis based on network and patent analysis
Source: PLoS One. 2023 Jan 12;18(1):e0279659. doi: 10.1371/journal.pone.0279659 (PMC9836267; doi:10.1371/journal.pone.0279659)

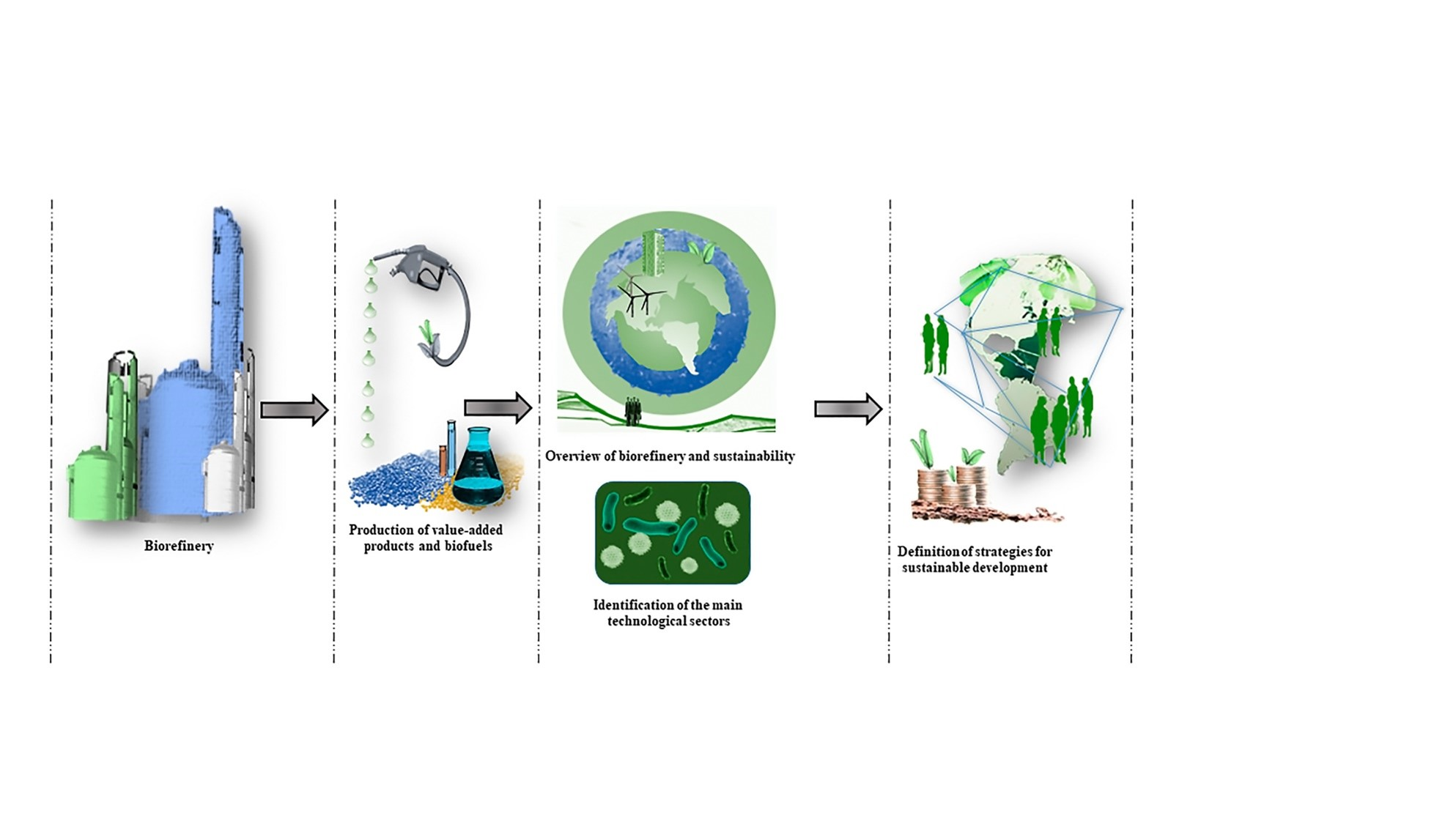

Supplement: S1 Graphical abstract — (TIF) [file pone.0279659.s004.tif]
